# Prognostic Value of Bladder Involvement in the Outcome of Upper Tract Urothelial Carcinoma

**DOI:** 10.3390/diagnostics13010153

**Published:** 2023-01-02

**Authors:** Sara Meireles, Nuno Dias, Diana Martins, Carolina Dias, Marina Gonçalves, João Silva, Carlos Martins Silva, Paulo Dinis Oliveira, Paula Soares, José Manuel Lopes

**Affiliations:** 1 Institute for Research and Innovation in Health (i3S), University of Porto, Rua Alfredo Allen 208, 4200-135 Porto, Portugal; 2 Institute of Molecular Pathology, Immunology of the University of Porto (IPATIMUP), Rua Júlio Amaral de Carvalho 45, 4200-135 Porto, Portugal; 3 Faculty of Medicine, University of Porto, Alameda Professor Hernâni Monteiro, 4200-319 Porto, Portugal; 4 Medical Oncology Department, Centro Hospitalar Universitário de São João (CHUSJ), Alameda Professor Hernâni Monteiro, 4200-319 Porto, Portugal; 5 Urology Department, Centro Hospitalar Universitário de São João (CHUSJ), Alameda Professor Hernâni Monteiro, 4200-319 Porto, Portugal; 6 Pathology Department, Centro Hospitalar Universitário de São João (CHUSJ), Alameda Professor Hernâni Monteiro, 4200-319 Porto, Portugal

**Keywords:** urothelial carcinoma, renal pelvis, ureter, bladder, outcome, prognostic factors

## Abstract

Accurately predicting the clinical prognosis of upper tract urothelial carcinoma (UTUC) seems crucial. We evaluated the effect of the involvement of urothelial bladder carcinoma (UBC) as a potential prognostic factor for overall survival (OS) and progression-free survival (PFS). The cohort included 115 patients with UTUC, subgrouped between January 2009 and December 2019 as follows: (1) only UTUC and (2) UTUC with synchronous or metachronous UBC (UTUC + UBC). Univariate and multivariate analyses were performed to identify independent prognostic factors for OS and PFS. Synchronous or metachronous UBC diagnosis in UTUC patients was an independent predictor of worse PFS (HR 3.326 CI 95% 1.474–7.503, *p* = 0.004), but it was not identified as a prognostic factor for OS (*p* > 0.05). Lymphovascular invasion (LVI) was associated with decreased PFS (HR 2.687 CI 95%1.172–6.163, *p* = 0.020) and OS (HR 4.980 CI 95%1.763–14.064, *p* = 0.002). This study indicates that concomitant or later UBC could predict a poor PFS, but it is not associated with a significantly worse OS in UTUC patients. The prognostic impact of LVI underlines its inclusion in the tumor staging system of UTUC.

## 1. Introduction

Upper tract urothelial carcinoma (UTUC) is a highly heterogeneous and aggressive disease. UTUC occurs in the pyelocaliceal system and ureter and comprises low-grade and high-grade tumors. These tumors represent up to 5–10% of all urothelial carcinomas, with most urothelial carcinomas being diagnosed in the bladder [1].

The five-year overall survival (OS) for UTUC patients is lower than that reported for urothelial bladder carcinoma (UBC) [2]. OS of UTUC may be only 35–50% in locally advanced disease and regional nodal involvement [3,4].

The management of UTUC is widely inferred from UBC, but both entities display some biological and genotypical divergent behaviors. Significant differences in the prevalence of common genomic alterations exist between UTUC and UBC [5]. Unlikely UBC, UTUC has also a higher microsatellite instability and loss of mismatch repair proteins (Lynch syndrome) [5]. A crucial unanswered question is whether temporally different UTUC and UBC tumors, developing in the same patient, with no previous UBC history, represent a genomic-related intravesical recurrence or a divergent primary tumor. The “monoclonality” and the “field cancerization” models address temporally related UTUC and UBC carcinogenesis [6,7]. The model that better reflects this biological situation continues to be undefined, but both mechanisms might be involved in developing UBC following previous UTUC [8,9,10,11].

The most common treatment for localized high-risk UTUC remains radical nephroureterectomy (RNU). At the same time, complete bladder-cuff removal, segmental ureterectomy and endoscopic procedures are suggested for non-invasive low-risk disease [5]. The effectiveness of neoadjuvant chemotherapy is still not well established, but adjuvant chemotherapy may increase OS in patients with UTUC [12].

Up to one-third of patients submitted to RNU will experience disease recurrence within five years [13] and the risk of developing a later UBC within two years is up to 22 to 47% [5,14]. Most bladder relapses are non-muscle-invasive, displaying low-grade, multiple, and papillary-like features [15,16]. However, the likelihood of subsequent UTUC following the diagnosis of a primary UBC is much lower (1–5%) [17]. For this context, the incidence of synchronous UBC is around 17% [18].

Accurately predicting the clinical prognosis of patients with UTUC is crucial. Until now, few studies have addressed the prognostic impact of synchronous or metachronous UBC diagnosis in the outcome of UTUC patients. Tumor stage and grade still represent the main prognostic factors, but controversy exists about the role of other clinical preoperative and pathological features [19,20,21].

The authors primarily aimed to evaluate the effect of the involvement of the bladder as a potential prognostic factor for OS and progression-free survival (PFS) in the outcome of these patients.

## 2. Materials and Methods

A retrospective, observational, single-center study was performed in Centro Hospitalar Universitário São João, between January 2009 and December 2019. The study comprised 129 adult patients (aged > 18 years) with high-grade and low-grade urothelial carcinoma of the pyelocaliceal system or ureter (UTUC). Inclusion criteria were: (a) conventional urothelial carcinoma; (b) mixed non-pure urothelial tumors with < 50 percent of variant histology features (glandular, squamous differentiation, sarcomatoid or micropapillary change); (c) concomitant carcinoma in situ. Patients with synchronous (within six months) or metachronous UBC diagnosis were included in this study. The cases were divided into two subgroups: (1) only UTUC; (2) UTUC with synchronous or metachronous UBC diagnosis (UTUC + UBC). Exclusion criteria were: (a) pure squamous, small cell, or sarcomatoid histologies; (b) previous UBC diagnosis; (c) patients with other active cancer.

All patients enrolled received appropriate preoperative clinical staging, including urethrocystoscopy to rule out concomitant UBC, computed tomography (CT)/magnetic resonance imaging (MRI), and flexible ureteroscopy with biopsy. The extent of regional lymph node dissection was based on preoperative images and intraoperative findings.

Demographic and clinicopathological baseline disease and patient features were collected in a database comprising a comprehensive review of all electronic medical records. The baseline clinical data assessment included: age at diagnosis, gender, risk factors, Eastern Cooperative Oncology Group (ECOG) performance status (PS) score, clinical presentation, tumor site and laterality, type of procedure, histologic type, tumor size, tumor grade, pathological stage, lymphovascular invasion (LVI), necrosis, concomitant carcinoma in situ and metastasis at diagnosis. The staging of cases followed the American Joint Committee on Cancer (AJCC) staging system. The tumor grading was assessed based on the World Health Organization (WHO) pathological grading system of malignant urothelial cancer in 2004.

Regular follow-up after RNU or kidney-sparing procedure was performed according to surveillance recommendations, with urine cytology and cystoscopy every three months for two years, every six months for the following three years, and then once annually. Abdominal and chest CT or MRI was suggested annually or more often, depending on the clinical stage.

The ethics regulatory hospital commission reviewed and approved the protocol (process nº 425/19). This study follows the recommendations of the Helsinki and Tokyo Declarations; the WHO and the European Community were respected.

### 2.1. Tumor Specimens

Representative hematoxylin and eosin slides from all archived formalin-fixed paraffin-embedded tumor samples concerning patients diagnosed with UTUC submitted to RNU resection or kidney-sparing approach was retrieved from the Department of Pathology. An expert genitourinary pathologist reviewed all the tissue samples to identify blocks containing adequate amounts of the tumor. The pathologists annotations included: histopathologic diagnosis features, namely variant histology, grade, pathological stage, concomitant carcinoma in situ, the presence and extent of tumor necrosis, and LVI.

### 2.2. Outcomes and Statistical Analysis

Subgroup descriptive statistics for all collected categorical variables were summarized as frequency counts (n) and percentages (%). Medians, minimum and maximum values were determined for continuous variables.

Statistical comparisons between relevant strata were made using appropriate statistical tests with significance reported as nominal *p*-values. *p*-values less than 0.05 were considered statistically significant.

The Chi-square test or Fisher’s exact test was used to compare categorical variables between groups. Independent samples *t*-test, when comparing two groups, was used to assess differences between patient subgroups for continuous variables.

PFS and OS were defined as the time between the date of diagnosis and disease progression or death, respectively. They were summarized using the Kaplan–Meier method according to different clinicopathological factors. Log-rank test was used to assess differences between subgroups.

Hazard ratio (HR) and 95% confidence intervals (CI) were calculated by the Cox regression model and adjusted for possible confounders for the survival analysis and prognostic factors identification. Statistical Package for Social Sciences (SPSS, IBM Corp, Chicago, IL, USA) software, version 26.0. was used for all statistical operative analyses.

## 3. Results

### 3.1. Baseline Clinicopathologic Characteristics

One hundred fifteen patients fulfilled the inclusion criteria for the final statistical analysis. All patients were Caucasian patients, and 69.6% were males. The median age at diagnosis was 75 years (41–94 years). Most patients had good PS with an ECOG score of 0–1 in 72.2% (n = 83). RNU was the standard approach in 92.2% of patients (n = 106). Forty-three patients had a history of synchronous (12.2%, n = 14) or metachronous UBC (25.2%, n = 29). Among them, 11% (n = 4) were diagnosed in the muscle-invasive stage, and three were treated with concomitant radical cystectomy.

Regarding the tumor-related features, the primary tumor location was predominantly in the renal pelvis in 57.4% of patients. According to the AJCC staging system, most had stage III (40.8%), and 12.2% had metastasis at diagnosis. A non-pure urothelial carcinoma with variant histology was presented in twelve patients (10.4%). Table 1 summarizes the clinicopathologic data in UTUC patients.

There were no statistically significant differences in defined variables across subgroups (only UTUC vs. UTUC + UBC), aside from the presence of metastatic disease at diagnosis (*p* = 0.013) (Table S1).These patients were excluded from the final statistical analysis of the proposed outcomes.

### 3.2. Survival Outcomes of UTUC and Differences between Both Subgroups (only UTUC vs. UTUC + UBC)

The median follow-up time was 19 months (0–130). Thirty-five patients had progression of the disease (35.4%), and the median time to progression was 11 months (range 0–100). Local progression was observed in 8% of patients (n = 8), bladder progression in 35.3% (n = 35) and distant metastasis was presented in 17% of patients (n = 17), with the median time to metastatic progression of 20 months (range 1–130). The mortality rate was 31.3% (n = 31), and twenty-one patients (21.2%) were lost to follow-up. The median metachronous UBC diagnosis was 10 months (3–61).

**Figure 1:**
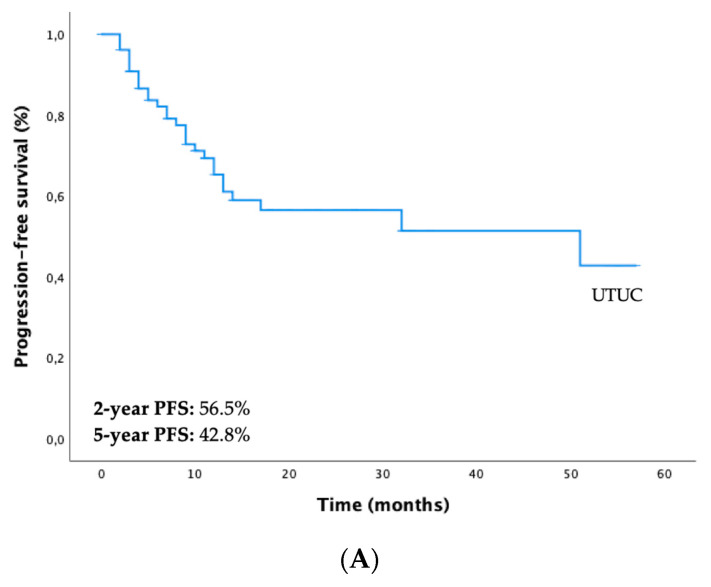

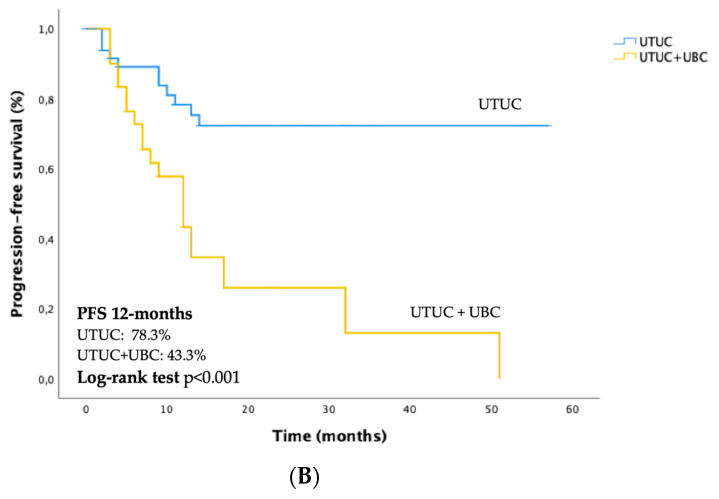
Kaplan–Meier curves for progression-free survival (PFS) of patients with UTUC (**A**) and stratified according to the two subgroups—only UTUC (blue line) vs. UTUC + UBC (yellow line) (**B**). Abbreviations: UBC—urothelial bladder cancer; UTUC—upper tract urothelial carcinoma.

**Figure 2:**
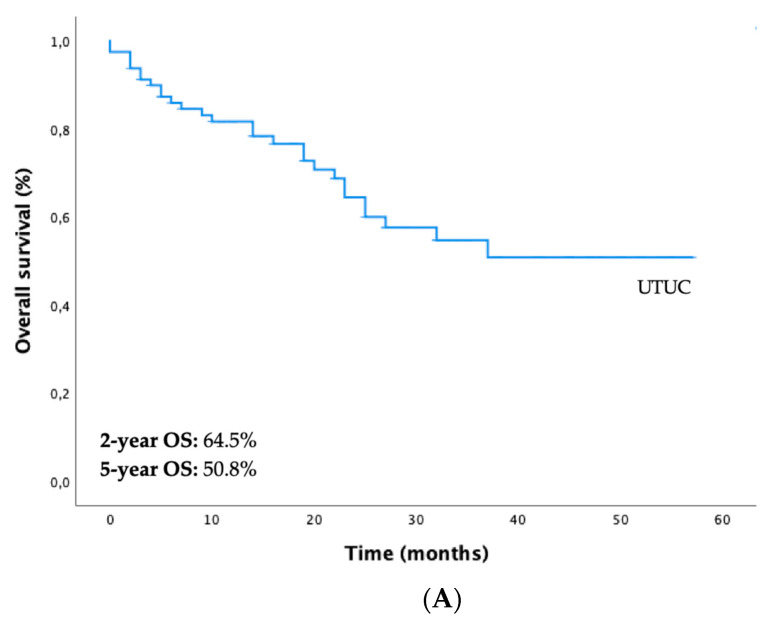

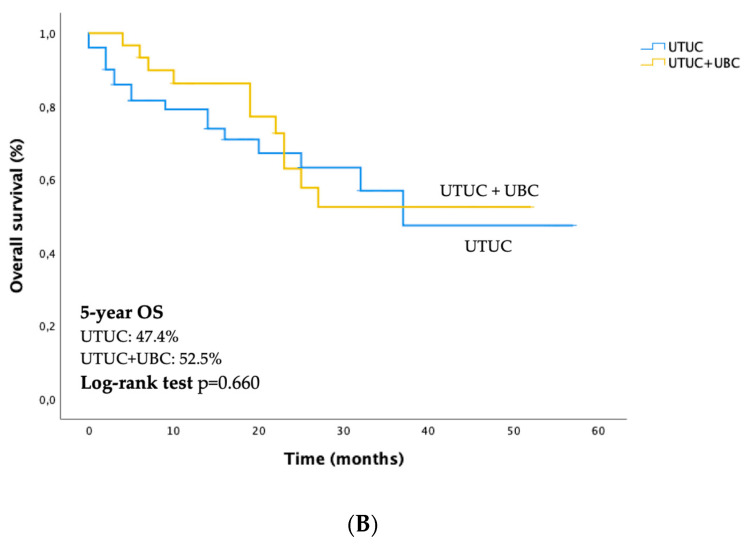
Kaplan–Meier curves for overall survival (OS) of patients with UTUC (**A**) and stratified according to the two subgroups—only UTUC (blue line) vs. UTUC + UBC (yellow line) (**B**). Abbreviations: UBC—urothelial bladder cancer; UTUC—upper tract urothelial carcinoma.

Regarding the whole study cohort, the 2-year and 5-year PFS were 56.5% and 42.8%, respectively (Figure 1A). There were statistically significant differences in PFS between both subgroups (PFS-12 months “only UTUC vs. UTUC + UBC” = 78.3% vs. 43.3%, *p* < 0.001) (Figure 1B). The 2-year and 5-year OS were 64.5% and 50.8%, respectively (Figure 2A). Differences in OS between both subgroups were not statistically significant (5-year OS “only UTUC vs. UTUC + UBC” = 47.4% vs. 52.5%, *p* = 0.660) (Figure 2B).

### 3.3. Prognostic Factors for PFS and OS and the Impact of Synchronous and Metachronous UBC Diagnosis in Outcomes

The histological subtype (*p* < 0.001), LVI (*p* = 0.001), AJCC staging system (*p* = 0.009), and presence of synchronous or metachronous UBC (*p* < 0.001) were significant prognostic factors for PFS (Table 2). The multivariate Cox regression analysis showed that LVI (HR 2.687 CI 95% 1.172–6.163, *p* = 0.020) and presence of synchronous or metachronous UBC (HR 3.326 CI 95% 1.474–7.503, *p* = 0.004) were independently associated with a worse PFS (Table 2).

ECOG PS ≥ 2 (*p* < 0.001), smoking status (*p* = 0.021), non-pure urothelial carcinoma histological subtype (*p* < 0.001), LVI (*p* < 0.001), AJCC stage (*p* = 0.02), presence of recurrence (*p* = 0.014) and distant metastasis (*p* < 0.001) were potential prognostic factors for OS on univariate analysis (Table 3). The multivariate Cox regression model, adjusting for a subset of clinically relevant variables, showed that ECOG PS ≥ 2 (HR 4.063 CI 95% 1.413–11.685, *p* = 0.009), smoking status (HR 5.060 CI 95% 1.584–16.165, *p* = 0.006), presence of LVI (HR 4.980 CI 95% 1.763–14.064, *p* = 0.002), and distant metastasis (HR 2.737 CI 95% 1.086–6.901 *p* = 0.033) were independent prognostic factors significantly associated with decreased OS (Table 3). Synchronous or metachronous UBC diagnosis was not a prognostic factor for worse OS on univariate and multivariate analysis (Table 3).

## 4. Discussion

The present study retrospectively reviewed a single institutional experience of UTUC patients to address the effect of synchronous and metachronous UBC diagnosis in their outcome. It also evaluated several other clinical and pathological factors that might predict disease progression and impact OS.

Over the last decade, a remarkable increase in the incidence of UTUC has been reported [22], but the prognosis remains poor. Given the rarity and heterogeneity of UTUC, data regarding prognostic factors for survival are scarce, and extrapolation from UBC has been used for risk stratification.

Primary urothelial carcinoma diagnosis is relevant for developing a subsequent tumor throughout the urinary tract, and patients with a primary UTUC have the highest probability of a UBC [18]. Until now, evidence regarding the approach of time-based association of UTUC and UBC diagnosis in the same patient has been omitted from current guidelines [5], and there is a lack of consensus concerning time-based cut-off points to define synchronous and metachronous bladder cancer. However, these events between UTUC and UBC represent an exclusive opportunity to understand time-based and anatomical biology and evaluate if bladder involvement could affect the natural history of UTUC.

Although the involvement of the bladder may stand for a higher risk for disease progression in UTUC patients, this study is the first to indicate that the association with bladder cancer does not seem to influence OS significantly. Our results suggest that the poor outcome of UTUC appears to be primarily related to the biological behavior of the upper tract tumor than associated with bladder cancer. Some reports show that previous or concomitant UBC is an independent predictor of lower recurrence-free survival and cancer-specific survival rates in patients with UTUC [23,24,25,26]. Nevertheless, the significance of bladder involvement in UTUC patients without previous UBC has been poorly evaluated, and data are currently sparse.

Our patients with UTUC, who underwent RNU, were under a thorough follow-up. According to the EAU guidelines, cystoscopy is mandatory in all patients diagnosed with a UTUC to rule out a concomitant UBC, as well as close surveillance with cystoscopy and urinary cytology during at least five years following surgical treatment [5]. These procedures might allow the detection of UBC in an earlier phase of the evolution of invasive carcinoma compared with a primary UBC diagnosis. Management of subsequent bladder cancer after RNU for UTUC is identical to a treatment strategy for a primary UBC [5]. However, recent data suggest that current primary UBC surgical guidelines are not entirely appropriate for UTUC-UBC patients [27].

UTUC patients usually exhibit a more aggressive disease and may not survive until the muscle-invasive bladder cancer stage is diagnosed. In the evolution of UTUC, local or distant metastasis is possibly more likely to develop than bladder tumor. As observed in our study, this presumption might explain a higher proportion of non-muscle invasive bladder cancer and a lack of significant impact of bladder tumor involvement on the outcome of UTUC, which seems to relate mainly to their unique anatomic and prognostic features.

The authors have also concluded that ECOG PS ≥ 2, tobacco consumption, LVI, and distant metastasis were associated with worse OS. LVI was also identified as an independent adverse prognostic factor for PFS.

The LVI has been linked with lymph node involvement and could represent a relevant step for tumor dissemination, predicting a poor prognosis [28,29,30]. Without lymph node involvement, the LVI may help identify patients at increased risk of recurrence, metastasis, and shorter cancer-specific survival despite radical surgery [31]. Still, the role of systemic lymphadenectomy during surgery for a UTUC remains controversial, leading to an uncertain pathologic lymph node status in most patients [32,33,34]. In our cohort, lymph node status was unknown in 73% of cases, consistent with previously reported low lymphadenectomy rates [31]. The results of our study support the significance of LVI as a prognostic factor and favor the recommendation to include LVI assessment in all pathology reports and staging systems of UTUC, which some authors have previously suggested [29]. These data also underline the need for a revision of the actual management of UTUC concerning lymphadenectomy and adjuvant chemotherapy indications.

Our study has some limitations, and these data should be interpreted cautiously. First, the study’s retrospective nature and apparent heterogeneity, with patients followed in a single tertiary center. Although one of the more extensive series in UTUC, the number of patients remains low, reflecting the rarity of the disease. Subgroups with smaller numbers may have contributed to the inability to show statistical significance. Additionally, the absence of molecular biomarkers data might reduce the study’s strength.

Literature data report that most UTUC and UBC sets have a clonal relatedness, despite notable differences between sequencing techniques in published series [2]. Thus, further molecular analysis should be conducted to clarify whether the tumor features of synchronous and metachronous UBC and UTUC are related or represent genetically unrelated primary tumors.

## 5. Conclusions

A better understanding of risk stratification and prognosis estimation of UTUC represents an undoubtful priority and should guide the selection of patients for more aggressive approaches and surveillance.

Our study reveals the aggressive clinical course of UTUC, reporting a high risk of recurrence and low OS.

The presence of concomitant or later UBC may be used to guide the management of UTUC, since it was associated with disease progression. However, it seems not related to a worse outcome.

Notably, our study strongly suggests the predictive risk of LVI, especially in lymph-node-negative or unknown-status patients, indicating that its inclusion in the tumor staging system may allow better management.

All in all, our study highlights the distinct biological behavior of UTUC and the relevance of synchronous and metachronous bladder cancer to understand biological and genotypical differences in both entities (UTUC and UBC). Further appropriately designed prospective studies should be conducted to validate our claims.

## Figures and Tables

**Table 1:** Baseline clinicopathologic characteristics of patients with upper tract urothelial carcinoma (UTUC).

Clinicopathologic Variables	Overall, n (%)
**Number of patients** (n/%)	115 (100)
**Age** (median, years)	75 (41–94)
**Gender** Male Female	80 (69.6) 35 (30.4)
**Risk factors** Smoking Occupational exposure	49 (55) 13 (25)
**ECOG PS** 0–1 ≥2	83 (72.2) 32 (27.8)
**Clinical presentation** Gross hematuria Hydronephrosis	67 (59.8) 56 (50)
**Laterality** Left Right	59 (51.3) 56 (48.7)
**Primary tumor location** Renal pelvis Ureter Both	66 (57.4) 33 (28.7) 16 (13.9)
**Surgical procedure** Nephroureterectomy Kidney-sparing approach	106 (92.2) 9 (7.8)
**Histological subtype** Pure UC Non-pure UC	103 (89.6) 12 (10.4)
**Lymphadenectomy** Yes No	32 (28.1) 82 (71.9)
**Tumor size, cm** ≤2 >2	16 (14) 98 (86)
**Multifocality** Yes No	11 (9.6) 104 (90.4)
**Tumor grade** Low-grade High-grade	7 (6.2) 106 (93.8)
**Lymphovascular invasion** Yes No	38 (33.9) 74 (66.1)
**Concomitant carcinoma in situ** Yes No	20 (17.4) 95 (82.6)
**Tumor necrosis** Yes No	16 (14.2) 97 (85.8)
**AJCC staging ^a^** 0is 0a I II III IV	2 (2) 7 (7.1) 25 (25.5) 20 (20.4) 40 (40.8) 4 (4.1)
**Lymph node involvement** Yes No	7 (22.6) 24 (77.4)
**Metastasis at diagnosis** Yes No	14 (12.2) 101 (87.8)
**Synchronous or metachronous UBC** Yes No	43 (37.4) 72 (62.6)

% valid percent; ^a^ excluding patients with metastasis at diagnosis and locally advanced unresectable disease; Abbreviations: AJCC—American Joint Committee on Cancer; cm—centimeter; ECOG PS—Eastern Cooperative Oncology Group Performance Status; n—number of patients; UBC—urothelial bladder carcinoma; UC—urothelial carcinoma; UTUC—upper tract urothelial carcinoma.

**Table 2:** Univariate and multivariate Cox regression analysis of prognostic factors for PFS.

**Variables**	Univariate Analysis			Multivariate Cox Regression Model		
	HR	CI 95%	*p*-Value	HRª	CI 95%	*p*-Value
**Gender** (male vs. female)	1.578	0.694–3.587	0.276	-	-	-
**ECOG PS** (0–1 vs. ≥2)	2.067	0.973–4.560	0.072	-	-	-
**Smoking status** (never vs former/current)	1.049	0.465–2.363	0.908	-	-	-
**Occupational exposure** (yes/no)	1.289	0.434–3.833	0.648	-	-	-
**Laterality** (left vs. right)	2.004	0.928–4.327	0.077	-	-	-
**Location of tumor**			0.945	-	-	-
Renal pelvis	0.983	0.286–3.382	0.979	-	-	-
Ureter	0.869	0.292–2.584	0.801	-	-	-
Both	1 (ref.)			-	-	-
**Hydronephrosis** (yes/no)	1.077	0.520–2.233	0.842	1.042	0.485–2.239	0.917
**Histological subtype** (pure UC vs. non-pure UC)	4.292	1.806–10.196	**<0.001 ^b^**	1.553	0.585–4.125	0.377
**Multifocality** (single vs. multiple)	1.909	0.725–5.025	0.190	-	-	-
**Tumor size** (≤2 vs. > 2)	3.005	0.711–12.692	0.134	-	-	-
**Tumor grade** (low-grade vs high-grade)	3.210	0.436–23.650	0.252	-	-	-
**Lymphovascular invasion** (absent vs. present)	3.598	1.722–7.520	**0.001 ^b^**	2.687	1.172–6.163	**0.020 ^b^**
**Carcinoma in situ** (absent vs. present)	1.430	0.609–3.354	0.411	-	-	-
**Tumor necrosis** (absent vs. present)	2.185	0.886–5.386	0.090	-	-	-
**AJCC staging system** (0is-0a-I vs. II-IV)	3.670	1.391–9.686	**0.009 ^b^**	2.216	0.767–6.403	0.142
**Lymph node involvement** (yes/no)	2.419	0.673–8.692	0.176	-	-	-
**Subgroups** (only UTUC vs. UTUC+UBC)	3.715	1.714–8.051	**0.001 ^b^**	3.326	1.474–7.503	**0.004 ^b^**

Abbreviations: AJCC—American Joint Committee on Cancer; CI: confidence interval; ECOG PS—Eastern Cooperative Oncology Group Performance Status; HR: hazard ratio; UBC—urothelial bladder carcinoma; UC—urothelial carcinoma; UTUC—upper tract urothelial carcinoma; ^a^ Adjusted for hydronephrosis, histological subtype, lymphovascular invasion and AJCC staging system; ^b^ Boldface values indicate statistical significance (*p* value < 0.05).

**Table 3:** Univariate and multivariate Cox regression analysis of prognostic factors for OS.

**Variables**	Univariate Analysis			Multivariate Cox Regression Model		
	HR	CI 95%	*p*-Value	HR^a^	CI 95%	*p*-Value
**Gender** (male vs. female)	2.439	0.926–6.426	0.071	-	-	-
**ECOG PS** (0–1 vs. ≥2)	3.637	1.700–7.783	**<0.001 ^b^**	4.063	1.413–11.685	**0.009 ^b^**
**Smoking status** (never vs. former/current)	3.571	1.214–10.506	**0.021 ^b^**	5.060	1.584–16.165	**0.006 ^b^**
**Occupational exposure** (yes/no)	1.227	0.427–3.525	0.704	-	-	-
**Laterality** (left vs. right)	1.156	0.546–2.449	0.705	-	-	-
**Location of tumor**			0.286	-	-	-
Renal pelvis	0.429	0.135–1.356	0.149			
Ureter	0.507	0.196–1.314	0.162			
Both	1 (ref.)					
**Hydronephrosis** (yes/no)	1.323	0.625–2.797	0.464	1.031	0.370–2.874	0.954
**Histological subtype** (pure UC vs. non-pure UC)	5.012	2.070–12.135	**<0.001 ^b^**	1.086	0.295–4.002	0.901
**Multifocality** (single vs. multiple)	1.038	0.313–3.442	0.952	-	-	-
**Tumor size** (≤2 vs. >2)	2.573	0.610–10.846	0.198	-	-	-
**Tumor grade** (low-grade vs high-grade)	1.190	0.161–8.808	0.865	-	-	-
**Lymphovascular invasion** (absent vs. present)	4.454	2.079–9.544	**<0.001 ^b^**	4.980	1.763–14.064	**<0.002 ^b^**
**Carcinoma in situ** (absent vs. present)	1.867	0.782–4.459	0.160	-	-	-
**Tumor necrosis** (absent vs. present)	1.508	0.521–4.364	0.448	-	-	-
**AJCC staging system** (0is-0a-I vs. II-IV)	3.168	1.201–8.360	**0.020 ^b^**	1.308	0.440–3.893	0.629
**Lymph node involvement** (yes/no)	3.093	0.887–10.782	0.076	-	-	-
**Subgroups** (only UTUC vs. UTUC+UBC)	1.185	0.553–2.537	0.663	1.009	0.394–2.586	0.985
**Recurrence^c^** (absent vs. present)	2.582	1.209–5.518	**0.014 ^b^**	-	-	-
**Local recurrence** (yes/no)	1.150	0.346–3.820	0.819	-	-	-
**Bladder recurrence** (yes/no)	2.433	0.576–10.282	0.227	-	-	-
**Distant metastasis** (yes/no)	3.920	1.842–8.342	**<0.001 ^b^**	2.737	1.086–6.901	**0.033 ^b^**

Abbreviations: AJCC—American Joint Committee on Cancer; CI: confidence interval; ECOG PS—Eastern Cooperative Oncology Group Performance Status; HR: hazard ratio; UBC—urothelial bladder cancer; UC—urothelial carcinoma; UTUC—upper tract urothelial carcinoma; ^a^ Adjusted for ECOG PS, smoking status, hydronephrosis, histological subtype, lymphovascular invasion, AJCC staging system and distant metastasis; ^b^ Boldface values indicate statistical significance (*p* value < 0.05); ^c^ Included patients with local recurrence, bladder recurrence or distant metastasis.

## Data Availability

The data presented in this study are available upon reasonable request from the corresponding author. The data are not publicly available due to patient privacy.

## Supplementary Materials

The following supporting information can be downloaded at: https://www.mdpi.com/article/10.3390/diagnostics13010153/s1, Table S1: Baseline clinicopathologic characteristics of both subgroups (only UTUC vs. UTUC + UBC).
