# Supplementary material for: Prognostic Value of Bladder Involvement in the Outcome of Upper Tract Urothelial Carcinoma
Source: Diagnostics (Basel). 2023 Jan 2;13(1):153. doi: 10.3390/diagnostics13010153 (PMC9818601; doi:10.3390/diagnostics13010153)
Supplement: Supplementary file 1 [file diagnostics-13-00153-s001.zip › Supplementary Materials - Word document.pdf]

Supplementary Materials

Table S1. Baseline clinicopathologic characteristics of both subgroups (only UTUC vs UTUC+UBC)

Characteristics	Overall, n (%)	Only UTUC, n (%)	UTUC+UBC, n (%)	P-value
**Number of patients** (n / %)	115 (100)	72 (62.6)	43 (37.4)	
**Age** (median, years)	75 (41-94)	73.5 (41-91)	77 (55-94)	**0.098**[a]
**Gender**				**0.971**[b]
Male	80 (69.6)	50 (69.4)	30 (69.8)	
Female	35 (30.4)	22 (30.6)	13 (30.2)	
**Risk factors**				
Smoking	49 (55)	31 (55.4)	18 (54.5)	**0.941**[b]
Occupational exposure	13 (25)	8 (24.2)	5 (26.3)	**0.560**[b]
**ECOG PS**				**0.398**[b]
0-1	83 (72.2)	50 (69.4)	33 (76.7)	
≥ 2	32 (27.8)	22 (30.6)	10 (23.3)	
**Clinical presentation**				
Gross hematuria	67 (59.8)	43 (60.6)	24 (58.5)	**0.833**[b]
Hydronephrosis	56 (50)	36 (50.7)	20 (48.8)	**0.844**[b]
**Laterality**				**0.257**[b]
Left	59 (51.3)	34 (47.2)	25 (58.1)	
Right	56 (48.7)	38 (52.8)	18 (41.9)	
**Primary tumor location**				**0.355**[b]
Renal pelvis	66 (57.4)	45 (62.5)	21 (48.8)	
Ureter	33 (28.7)	18 (25)	15 (34.9)	
Both	16 (13.9)	9 (12.5)	7 (16.3)	
**Surgical procedure**				**0.077**[c]
Nephroureterectomy	106 (92.2)	69 (95.8)	37 (86)	
Kidney-sparing approach	9 (7.8)	3 (4.2)	6 (14)	
**Histological subtype**				**0.512**[b]
Pure UC	103 (89.6)	64 (88.9)	39 (90.7)	
Non-pure UC	12 (10.4)	8 (11.1)	4 (9.3)	
**Lymphadenectomy**				**0.689**[b]
Yes	32 (28.1)	19 (26.8)	13 (30.2)	
No	82 (71.9)	52 (73.2)	30 (69.8)	
**Tumor size, cm**				**0.984**[b]
≤ 2	16 (14)	10 (14.1)	6 (14)	
> 2	98 (86)	61 (85.9)	37 (86)	
**Multifocality**				**0.326**[c]
Yes	11 (9.6)	5 (6.9)	6 (14)	
No	104 (90.4)	67 (93.1)	37 (86)	
**Tumor grade**				**0.540**[c]
Low-grade	7 (6.2)	4 (5.7)	3 (7)	
High-grade	106 (93.8)	66 (94.3)	40 (93)	
**Lymphovascular invasion**				**0.141**[b]
Yes	38 (33.9)	27 (39.1)	11 (25.6)	
No	74 (66.1)	42 (60.9)	32 (74.4)	
**Concomitant carcinoma in situ**				**0.808**[b]
Yes	20 (17.4)	13 (18.1)	7 (16.3)	
No	95 (82.6)	59 (81.9)	36 (83.7)	
**Tumor necrosis**				**0.612**[b]
Yes	16 (14.2)	9 (12.9)	7 (16.3)	
No	97 (85.8)	61 (87.1)	36 (83.7)	
**AJCC staging***				**0.777**[c]
0is	2 (2.0)	2 (3.5)	0	
0a	7 (7.1)	4 (7.0)	3 (7.3)	
I	25 (25.5)	15 (26.3)	10 (24.4)	
II	20 (20.4)	10 (17.5)	10 (24.4)	
III	40 (40.8)	23 (40.4)	17 (41.5)	
IV	4 (4.1)	3 (5.3)	1 (2.4)	

**Lymph node involvement**				**0.053[c]**
Yes	7 (22.6)	7 (35)	0	
No	24 (77.4)	13 (65)	11 (100)	
**Metastasis at diagnosis**				**0.013[b]**
Yes	14 (12.2)	13 (18.1)	1 (2.3)	
No	101 (87.8)	59 (81.9)	42 (97.7)	

% valid percent;

*excluding patients with metastasis at diagnosis and locally advanced unresectable disease;

Abbreviations: AJCC – American Joint Committee on Cancer; cm - centimeter; ECOG PS – Eastern Cooperative Oncology Group Performance Status; n – number of patients; UBC – urothelial bladder cancer; UC – urothelial carcinoma; UTUC – upper tract urothelial carcinoma

[a] Student's t-test

[b] Chi-square test

[c] Fisher's exat test
